# Conductance stability and Na+ interaction with Shab K+ channels under low K+ conditions

**DOI:** 10.1080/19336950.2021.1993037

**Published:** 2021-10-26

**Authors:** Froylán Gómez-Lagunas, Elisa Carrillo, Carolina Barriga-Montoya

**Affiliations:** School of Medicine, Department of Physiology, National Autonomous University of Mexico (Unam), México City, México

**Keywords:** Potassium channels, hypokalemia, conductance stability, selectivity, TEA blockage, Na blockage

## Abstract

K^+^ ions exert a structural effect that brings stability to K^+^ selective pores. Thus, upon bathing Shab channels in 0 K^+^ solutions the ion conductance, G_K_, irreversibly collapses. Related to this, studies with isolated KcsA channels have suggested that there is a transition [K^+^] around which the pore takes one of two conformations, either the low (non-conducting) or high K^+^ (conducting) crystal structures. We examined this premise by looking at the K^+^-dependency of G_K_ stability of Shab channels within the cell membrane environment. We found that: K^+^ effect on G_K_ stability is highly asymmetrical, and that as internal K^+^ is replaced by Na^+^ G_K_ drops in a way that suggests a transition internal [K^+^]. Additionally, we found that external permeant ions inhibit G_K_ drop with a potency that differs from the global selectivity-sequence of K^+^ pores; the non-permeant TEA inhibited G_K_ drop in a K^+^-dependent manner. Upon lowering internal [K^+^] we observed an influx of Na^+^ at negative potentials. Na^+^ influx was halted by physiological external [K^+^], which also restored G_K_ stability. Hyperpolarized potentials afforded G_K_ stability but, as expected, do not restore G_K_ selectivity. For completeness, Na^+^ interaction with Shab was also assessed at depolarized potentials by looking at Na block followed by permeation (pore unblock) at positive potentials, in solutions approaching the 0 K^+^ limit. The stabilizing effect of negative potentials along with the non-parallel variation of Na^+^ permeability and conductance-stability herein reported, show that pore stability and selectivity, although related, are not strictly coupled.

## Introduction

In addition to permeate through potassium channels K^+^ ions keep stable the pore conformation capable to conduct ions. Evidence of this central property of K^+^ channels came first from electrophysiological studies carried out on voltage-gated channels. Studying the squid K channel Almers & Armstrong [1] noticed that K channel activity gradually vanishes upon K^+^ removal from the recording solutions. A similar phenomenon was afterward observed in Shaker channels [2]. It was found that, in contrast to the squid channel, the Shaker K^+^ conductance (G_K_) remains unchanged upon exposure to 0 K^+^ solutions, as long as the channels are kept undisturbed, closed, while they are bathed in 0 K^+^, however if they are gated, with the delivery of depolarizing pulses, then G_K_ collapses. In the case of Shaker, G_K_ drop does not depend on the frequency of pulsing in 0 K^+^, and is fully reversed by prolonged depolarizations, which demonstrates that the drop of Gk is not due to inactivation [2]. Shab responds to immersion in 0 K^+^ solutions in a manner similar to that of the squid K^+^ channel, namely: Shab G_K_ drops passively (i.e., with the channels kept closed at the resting potential) during channel exposure to 0 K^+^, and the drop of G_K_ is irreversible [1,3].In both Shab and Shaker channels, Na^+^ replacement with the bigger and impermeant choline or NMG ions slows down G_K_ collapse in 0 K^+^, although interestingly, this effect is more pronounced in Shaker than in Shab [3–5]. Na^+^ substitution by Cs^+^, in the absence of K^+^, also maintains the Shab conductance stable [6]. These observations indicate that Na^+^ ions somehow catalyze G_K_ collapse in the absence of K^+^. G_K_ collapse in 0 K^+^ is voltage dependent, hyperpolarized holding potentials inhibit G_K_ drop of both Shaker and Shab channels [2,5,7,8]. The latter shows that the membrane potential has a relevant role on the K^+^-dependent G_K_ stability, one that cannot be assessed by crystal structures nor biochemical studies carried out with purified, membrane-devoid, proteins.Regarding the structural framework of the above observations, the known landmark studies of MacKinnon´s lab [9], have shown that K^+^ channel pores present 4 K^+^ binding sites (named s1 to s4, from outside to inside) placed in series and at regular distances, along the narrow (3 Å radius x 12 Å long) and extracellularly oriented region of the pore known as the selectivity filter (SF), which contains the amino-acid signature sequence of K^+^ channels (TVGYG) [9,10]. Oxygens of main-chain carbonyls of signature sequence amino acids, along with that of the hydroxyl side-chain of the s4 threonine, point toward the pore lumen, forming 4 cages of 8 oxygens each, that coordinate a centrally located K^+^ ion (radius = 1.3 Å). In the zero-voltage crystals, K^+^ ions are located at every third position, with an s1-s3 occupancy being as likely as an s2-s4 K^+^ occupancy. With the cytoplasmic gate closed, an additional K^+^ ion is seen at the center of the region known as the central cavity of the pore [9]. The above, capable to conduct K^+^ ions structure, is obtained with high [K^+^] crystallization solutions (200 mM), and hence it is referred to as the high K^+^ structure, to contrast it against a KcsA structure observed with low K^+^ crystallization solutions (5 mM), named the low K^+^ structure [11]. The latter, presents a distorted pore structure, considered unlikely to conduct K^+^, in which the SF harbors a single K^+^ ion, located at either s1 or s4, since the SF valine and glycine residues have a distorted orientation, which eliminates the central s2 & s3 positions as K^+^ binding sites. Related to this, and interestingly, it has been proposed that there is a transition [K^+^], around which the pore falls into either the low or high-K^+^ conformations [12–14].On the other hand, in spite of the mentioned physiological and crystallographic observations, not all K^+^-selective channels seem to require K^+^ ions to remain functional. For example, it has been reported that delayed rectifier channels of frog sympathetic neurons [15], as well as Kv2.1 channels of cervical ganglia [16] remain active upon K^+^ removal, although allowing the anomalous passage of Na^+^ ions. Further work is clearly needed to determine the K^+^ requirements of the K^+^ channel family.

Herein, we extend our former observations on the K^+^-dependent stability of K^+^ channels. We gradually replaced K^+^ with Na^+^ ions, and observed the effect of this manipulation in stability, selectivity, and Na^+^ block of Shab G_K_. Among other observations we report that, with channels within the cell membrane environment, as internal [K^+^] decreases G_K_ drops in a way consistent with the presence of a transition [K^+^_i_], around which the pore either falls relatively fast into an irreversible non-conducting state, or remains comparatively longer in a capable to conduct, although less selective, conformation; in qualitative agreement with studies carried out with isolated KcsA channels. Additionally, we found that along with G_K_ destabilization, hypokalemic (non-zero K^+^) solutions allow Na^+^ permeation at negative voltages. Hyperpolarized holding potentials keep channel conductance stable under these conditions [7] but, as expected, they do not restore pore selectivity. On the other hand, at depolarized potentials we found that Na^+^ interaction with the channels (i.e., internal Na^+^ block with solutions that approach the 0 K^+^ limit) becomes apparent from negative potentials, and presents a blockage electrical-distance near one. The differential role of the membrane voltage on stability and selectivity, along with the non-parallel variation of G_K_ stability and Na^+^ permeability herein reported, support the notion that: pore stability and selectivity, although clearly related [e.g., 6, 8, 17, 18, this work] are not strictly coupled parameters.

## Materials and methods

### Cell culture and Shab channel expression

Insect Sf9 cells were grown at 27°C in Ex-Cell 420 medium (Sigma) containing an added 10% fetal bovine serum (Sigma). Cells were infected with a recombinant baculovirus containing Shab K-channel cDNA (dShab 11, Gene Bank Accession Number M32659.1) [17, 18] with a multiplicity of infection ~10. Experiments were conducted 48 h after infection, as reported [7].

### Electrophysiological recordings

Macroscopic currents were recorded under whole-cell patch clamp, with an Axopatch 1D amplifier (Axon Instruments). Currents were filtered on-line and sampled at rates that fulfilled the Nyquist criteria, with a Digidata 1322A interface (Axon Instruments). Electrodes were made of borosilicate glass (KIMAX 51) pulled to a 1–1.5 MΩ resistance, 80% of series resistance was compensated, as reported [7].

### Solutions

Solutions will be named by their main active, test, cation and location across the membrane. External solutions contained (mM): Na_o_ (0 K_o_): 145 NaCl, 10 CaCl_2_, 10 HEPES-NaOH. XK_o_: X KCl, 145-X NaCl, 10 CaCl_2_, 10 HEPES-NaOH. Internal solutions contained (mM): K_i_: 30 KCl, 90 KF, 2 MgCl_2_, 10 EGTA-KOH, 10 HEPES-KOH; Na_i_: 30 NaCl, 90 NaF, 2 MgCl_2_, 10 EGTA-NaOH, 10 HEPES-NaOH. X_Ki_ solutions were prepared by mixing K_i_ and Na_i_ solutions to yield the desired [K^+^_i_], keeping the molar fractions X_Ki_+X_Nai_ = 1. pH of all solutions was 7.2. Solutions were changed using a gravity driven perfusion system, as reported [7]. With a recoding chamber volume of 250 μL, full exchange of solutions took< 10 sec. Junction potentials estimated following standard procedures, as reported [6,19], were smaller than ±3 mV.

### Data analysis

Currents were analyzed with Clampfit (Axon Instruments). Curve fitting was carried out with SigmaPlot 10.6 & GraphPad Prism 5 (GraphPad software). Statistical analysis was done with GraphPad Prism 5. Points are mean ±SEM of at least 4 independent experiments. Significance level was set at 0.05.

## Results

**Figure 1.**
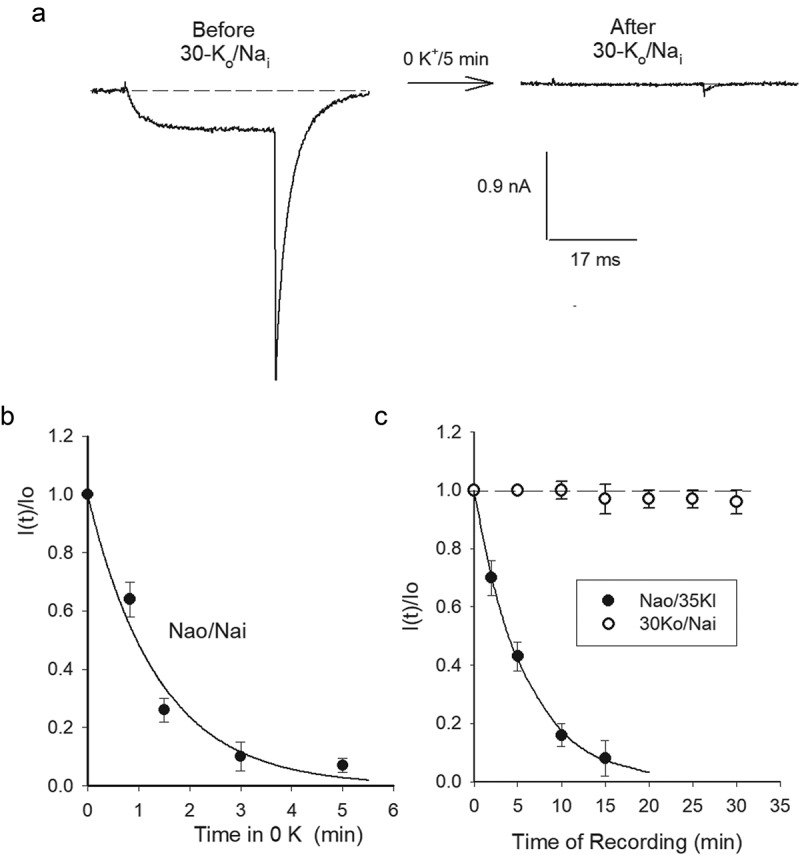
G_K_ drop in hypokalemic solutions. (a) Left panel, Control I_K_ evoked by a 0 mV/30 ms pulse with the cell bathed in 30K_o_/Na_i_ solutions. HP = −80 mV. Right panel, I_K_ left after exposing the cell to 0 K^+^ (Na_o_/Na_i_) solutions across the membrane for 5 min (see Text), while V_m_ was kept constant at the HP. (b) I(t)/I(0) vs. Time in 0K^+^, I(t) is I_K_ left after exposing the cell for t-min to 0 K^+^, I_o_ is the initial, control, I_K_, as in A. The line is the fit of the points with a single exponential function I(t)/I(0) = exp(-t/Ʈ), with Ʈ = 1.4 min. (c) G_K_ stability assessed as I(t)/I(0) vs. Time of whole-cell recording. I_K_ was measured applying 0 mV/30 ms activating pulses at the indicated times, V_m_ = −80 mV between pulses. Cells were bathed in either Na_o_/35K_i_ (closed circles) or 30K_o_/Na_i_ (open circles) solutions. The solid line is the fit of the points (Na_o_/35K_i_) with a single exponential function with Ʈ = 5.7 min, as in B. A&B were taken from Gomez-Lagunas, 2007 [7]

For a reference, a typical experiment that illustrates the irreversible loss of K^+^ conductance, G_K_, upon channels exposure to 0 K^+^ solutions is presented in (Figure 1(a)). The experiment was carried out using a 30 mM [Ko^+^] control solution (see Methods), a concentration which is near the hemolymph [K^+^] concentration of Drosophila (36–55 mM) [20], the organism from which Shab DNA was cloned [17], and that, more importantly, affords stability to Shab G_K_ (see below). To test G_K_ stability, control K^+^ current (Figure 1(a) left panel) is recorded by applying activating pulses (0 mV/30 ms from the HP of −80 mV), with the cell bathed in solutions where G_K_ is stable (30 K_o_/Na_i_, see Methods). Thereafter, the cell is superfused with the test, Na^+^-containing, external solution lacking K^+^ (Na_o_/Na_i_ condition) for a variable time (5 min in the case illustrated), with the channels kept closed at the HP (indicated by the arrow). Finally, the cell is superfused back with 30 mM [K_o_] solution, and the state of the channels is tested. The trace in the right panel illustrates that a 5 min exposure to 0 K^+^ eliminates G_K_ almost completely. GK stability is assessed from experiments as in A by the ratio I(t)/I_o_ (Figure 1(b)), where I(t) is I_K_ amplitude left after t-min exposure of channels to 0 K^+^, and I_o_ is the control (t = 0) current amplitude (data from [7], see Figure Legend).

We had previously stated that external K^+^ was slightly more effective at keeping G_K_ stable than internal K^+^. However, this was observed comparing the effect of high [K^+^] solutions, which already kept G_K_ rather, and comparably, stable (120 mM K_i_ vs. 100 mM K_o_) [3]. Since the sidedness of K^+^ effect on G_K_ has a clear relevance to K^+^ channels functioning, we reviewed our previous statement by testing the relative effectiveness of lower, nearly symmetric, [K^+^] across the membrane. The latter is presented in (Figure 1(c)) which shows G_K_ stability as a function of the time of whole-cell recording, with channels bathed in either 30K_o_/Na_i_ (as the control in A, open circles) or Na_o_/35 K_i_ solutions (closed circles, see Figure legend). Note that, G_K_ is notably more stable with K^+^ present in only the external solution (see later & Discussion).

### G_K_ stability under low [K^+^] conditions. A test for a transition [K_i_^+^]

Studies with purified KcsA proteins have suggested that there is a transition [K^+^] around which the pore takes either the low or the high-K^+^ crystal structures that have been observed in this channel (Introduction). As these studies are done with isolated proteins, we were interested in determining whether in the cell membrane environment there was also evidence of a corresponding transition [K_i_], around which the pore would either fall relatively fast into an irreversible non-conducting state, or remain comparatively longer in a functional conformation.

**Figure 2.**
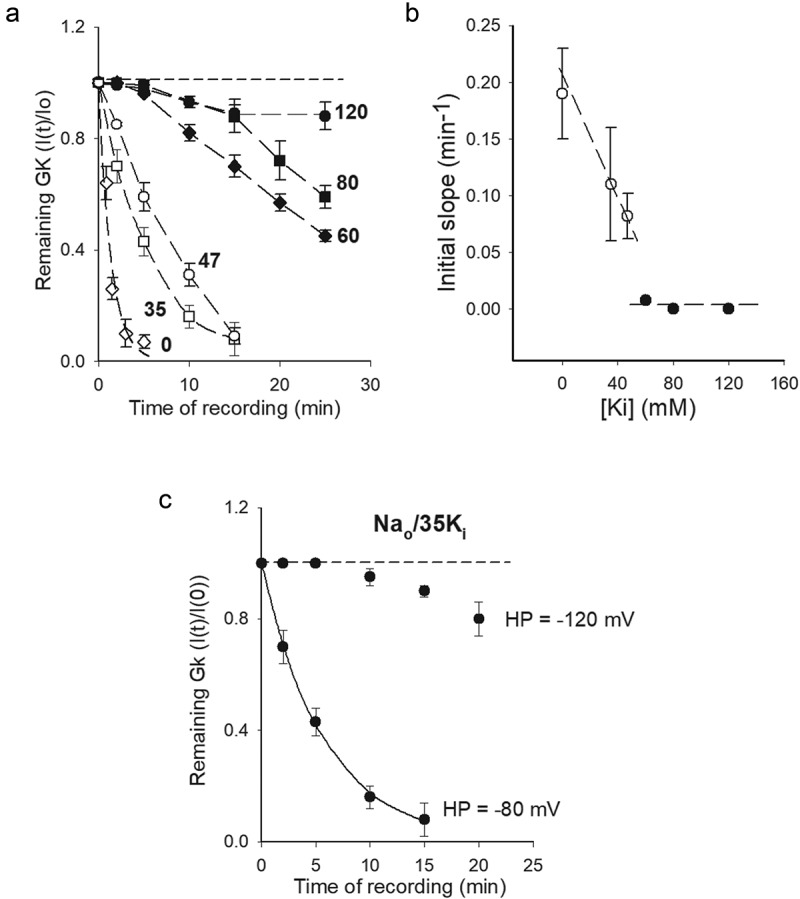
G_K_ stability vs. [K_i_]. (a) Remaining G_K_ (I(t)/I_o_) vs Time of recording, as in Figure 1c. Cells were bathed in Na^+^-containing internal solutions containing the indicated [K_i_] (with X_Ki_+X_Nai_ = 1, see text). External solution was Na_o_. Note that the data clusters into two sets. (b) Initial slope vs. [K_i_]. Initial slope was assessed as the slope of the straight line that connects the first two points in A (I(t = 0) with the next I(t ≈ 2 min) point. The dashed lines have no physical meaning, they serve to best point out the data division into two sets. (c) G_K_ stability of channels bathed in Na_o_/35K_i_ solutions at the HP of either −80 or −120 mV, as indicated

To test the above, G_K_ stability was assessed in the absence of external K^+^, following the time course of I_K_ amplitude in cells dialyzed with, Na^+^-containing, internal solutions having a variable [K^+^_i_] (with X_Ki_+X_Nai_ = 1, where X stands for molar fraction, see Methods), as in (Figure 1(c)). Figure 2a shows G_K_ stability (I(t)/I_o_ ratio) plotted against time of whole-cell recording, at the indicated [K^+^_i_]. Note that, as expected, G_K_ stability drops as internal [K^+^] decreases and, more interestingly, note that the data clusters into two groups, with markedly different rate of G_K_ drop, one corresponding to [K^+^_i_]≤ 47 mM, and the other to [K^+^_i_]≥ 60 mM. We interpret this observation as suggestive of a transition [K^+^_i_], located somewhere between 47 and 60 mM, at −80 mV. The latter is more clearly noticed in (Figure 2(b)) which presents the plot of the initial slope of the points in A vs. [K^+^_i_] (see Figure legend). Note the abrupt change in the slopes trend. The dashed lines through the points serve to enhance the markedly different trend of the slopes, and lack any physical meaning (see (Figure (6)) of Zhou & Mackinnon, 2003 [14]). The above observations qualitatively agree with the studies done with purified KcsA channels [11–14], suggesting that similar K^+^-dependent structural changes may occur in the pore of voltage-dependent channels within the cell membrane environment, although in the latter G_K_ stability is voltage-dependent (see below & Discussion).

Figure 2(c) shows, that similar to the case of 0 K^+^ across the membrane [7], a hyperpolarized HP (−120 mV) stabilizes G_K_ in hypokalemic, non-0 K^+^, conditions. This shows that even millimolar amounts of internal K^+^ do not bypass the stabilizing role exerted by negative V_m_.

### Inhibition of G_K_ drop by external monovalent cations

Thereafter, we studied the effect of externally added cations on G_K_ stability. Our goal was to determine whether the potency with which different ions may prevent G_K_ collapse correlated with the global selectivity-sequence of K^+^ pores.

**Figure 3.**
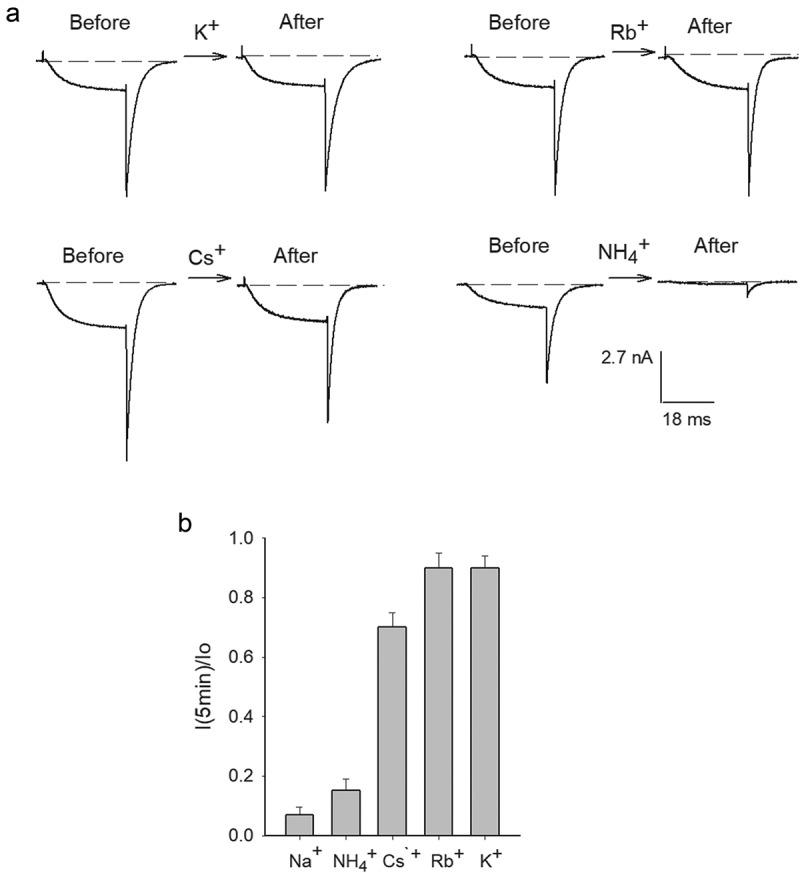
Inhibition of G_K_ drop by externally added monovalent cations. (a) Representative I_K_ recorded in 30K_o_/Na_i_ solutions, before (left traces) and after (right traces) bathing the cells for 5 min in Na_o_ solution containing the indicated test cation ([test cation] = 5 mM). V_m_ was kept constant at −80 mV during cells exposure to test solutions. (b) Remaining I_K_ (I_K_(t = 5 min)/I_o_) after a 5-min exposure to the indicated solutions. Effects of Rb^+^ (0.9 ± 0.05) and K^+^ (0.9 ± 0.04) are not statistically different

**Figure 4.**
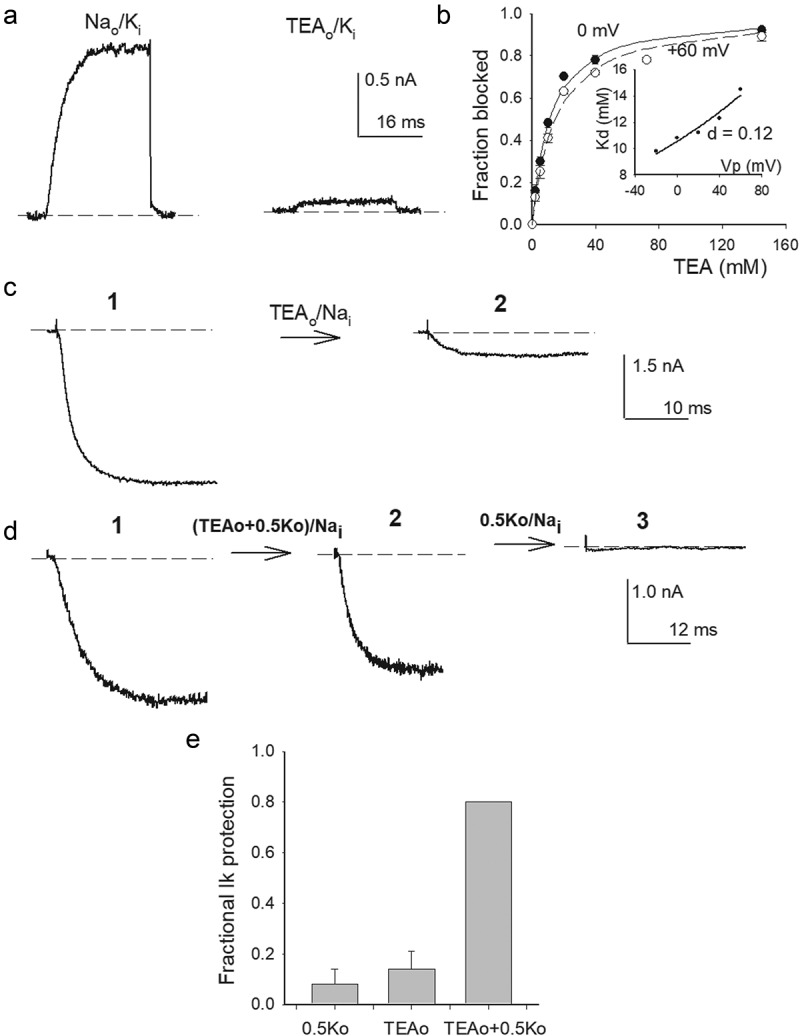
TEA_o_ block and inhibition of G_K_ drop. (a) Left panel, control I_K_ evoked by a 0 mV/30 ms pulse applied from −80 mV in Na_o_/K_i_ solutions. Right panel, I_K_ recorded upon bathing the same cell with 145 mM TEA_o_ solution (see Methods), ~80% of I_K_ was blocked. (b) Fractional TEA_o_ block vs. [TEA_o_]. Fraction blocked = 1-(I([TEA])/I_o_), where I([TEA]) is I_K_ amplitude in the presence of the indicated [TEA], I_o_ is the corresponding control I_K_. Closed circles: test pulse = 0 mV; open circles: test pulse = +60 mV. The lines are the fit of the points with a Michaelis-Menten equation with K_d_(0 mV) = 10.8 mM, or K_d_(+60 mV) = 14.5 mM. Inset: K_d_ vs. V_m_. K_d_s are the best fit parameters of the corresponding Michaelis-Menten curves. The line is the fit of the points with a Woodhull equation: K_d_(V_m_) = K_d_(0)*exp[zd(F/RT)*V_m_]; with parameters d = 0.12, K_d_(0 mV) = 10.5 mM, z = +1; F,R,T have their usual meaning. (c) TEA_o_ inhibition of G_K_ collapse in 0 K^+^. Left panel: Control I_K_ evoked by a 0 mV/30 ms pulse, applied from −80 mV, in 30K_o_/Na_i_ solutions (labeled 1). Right panel: I_K_ left after immersing the cell in, 0 K^+^, TEA_o_ solution (TEAo/Nai) for 5 min, with Vm constant at −80 mV (trace labeled 2, see Methods). (d) TEA_o_ inhibition of G_K_ collapse in the presence of 0.5 mM K_o_^+^. Left panel: Control I_K_ recorded as in C (labeled 1). Middle panel: I_K_ left after superfusing the cell for 5 min with TEA_o_ solution containing 0.5 mM [K_o_^+^], with Vm at −80 mV (labeled 2). Right panel: I_K_ left after the subsequent exposure of the same cell in 2 to Na_o_ (0 TEA) solution, containing 0.5 mM [K_o_^+^], at −80 mV (labeled 3). (e) Fractional I_K_ protection after bathing the cells for 5 min in the indicated solutions

Figure 3(a) illustrates I_K_ recorded in 30K_o_/Na_i_ solutions before and after bathing the cells for 5-min with the indicated test cation, Na^+^-containing, external solutions ([test cation] = 5 mM), while V_m_ was kept constant at the HP of −80 mV, as in (Figure 1(a)). The observations, summarized in (Figure 3(b)), show that monovalent cations prevent G_K_ drop with a potency that follows the sequence: Na^+^≪NH_4_^+^≪Cs^+^<Rb^+^≈K^+^. The latter differs from the global selectivity-sequence of K channels, in which NH_4_^+^ is more permeable than Cs^+^. This suggests the simplest hypothesis that the order in (Figure 3(b)) could correspond to the selectivity of a particular, externally accessible, pore site which upon occupancy by a suitable cation keeps G_K_ stable. Regarding the test cation concentration in (Figure 3(a)) (5 mM), it is interesting to note that although the Drosophila hemolymph presents an elevated [K^+^], due to its blood-brain barrier a much lower 5 mM [Ko^+^] baths brain cells [20], similar to the external [Ko^+^] of mammalian cells. Considering the observations in (Figure 3), and that the non-permeant TEA cation inhibits G_K_ drop of Shaker acting from the external side of the membrane [2], we seek to determine whether external TEA would also inhibit Shab G_K_ drop. To do this, firstly we determined the basic parameters of TEA_o_ block of Shab, namely the voltage dependence of its apparent affinity, which as far as we know has not been reported yet. The traces in (Figure 4(a)) indicate that ~80% of channels are blocked by 145 mM TEA_o_, at 0 mV. The average dose-response curve of experiments as in A (Figure 4(b)) indicates that TEA_o_ blocks Shab with a least-squares K_d_ of 10.8 mM at 0 mV, and 14.5 mM at +60 mV, both obtained by fitting the points with a Michaelis-Menten curve (lines through the points).

The latter is best appreciated in the inset which shows the plot of least-squares K_d_ vs. V_m_. Notice that TEAo affinity decreases with voltage, as expected. The line is the fit of the points with a standard Woodhull equation [21], with parameters K_d_(0 mV) = 10.8 mM, and electrical distance d = 0.12 which agrees with a superficially located TEA_o_ binding site (see Figure legend). Once the basic parameters of TEA_o_ block were determined, its effect on G_K_ stability was studied (Figure 4(c)) following the protocol in (Figure 1(a)), namely: Control I_K_ was recorded in 30K_o_/Na_i_ solutions (trace labeled 1) and thereafter the cell was superfused for 5 min with 145 mM TEA, 0 K^+^, external solution (indicated by the arrow), finally the cell was superfused back with 30Ko solution, and the state of the channels was tested. The trace in the right panel (labeled 2) shows that TEAo inhibited Gk drop (compare against Figure 1(a)), but in an extent smaller than that expected considering its blockage affinity (~15% of channels remained active).

After having observed the above result, we considered that it had been reported that mammalian Kv2.1 channels cease to be blocked by TEA_o_ upon K^+^ removal, and that micromolar amounts of external K^+^ restore block [22] (see Discussion). Therefore, we tested again the effect of TEA_o_ on G_K_ drop (Figure 4(d)), but this time adding 0.5 mM K_o_^+^ to the test TEA_o_ solution (0.5 K^+^+145 TEA_o_/Na_i_). A comparison of control I_K_ (left panel, labeled 1) against I_K_ left after bathing the cell for 5 min with 0.5K+TEAo solution (middle panel, labeled 2) shows that this time TEAo inhibited Gk drop in an extent (~75%) near to that expected from its apparent block affinity in standard recording conditions.

Finally, to check for the possibility that the increased G_K_ protection could have been exerted by the co-added 0.5 mM K^+^, immediately after middle I_K_ was recorded, the cell was superfused for 5 minutes with a 0.5 mM K_o_ solution lacking TEA (0.5K_o_/Na_i_). The trace in the right corner (labeled 3) shows that 0.5 mM K_o_^+^ by itself is unable to preserve G_K_ (see Discussion). These observations are summarized in (Figure 4(e)). Note that 145 mM TEA_o_ (i.e., TEA_o_+0.5K_o_) effectively protected G_K_ (~80%), in near quantitative agreement with its expected extent of block. TEAo blocks Shab with electrical distance of 0.12, consistent with TEAo binding right above the selectivity filter as expected [23,24]. Hence, the observations in (Figures 1b, 3 & 4) indicate that (1) placing a suitable ion at the extracellular entry of the pore keeps G_K_ quite stable, and (2) rule out the possibility that TEA_o_ inhibition of G_K_ drop might be due to its trapping of K^+^ ions within the pore (see Discussion).

### Na^+^ permeation under low [K^+^] conditions

**Figure 5.**
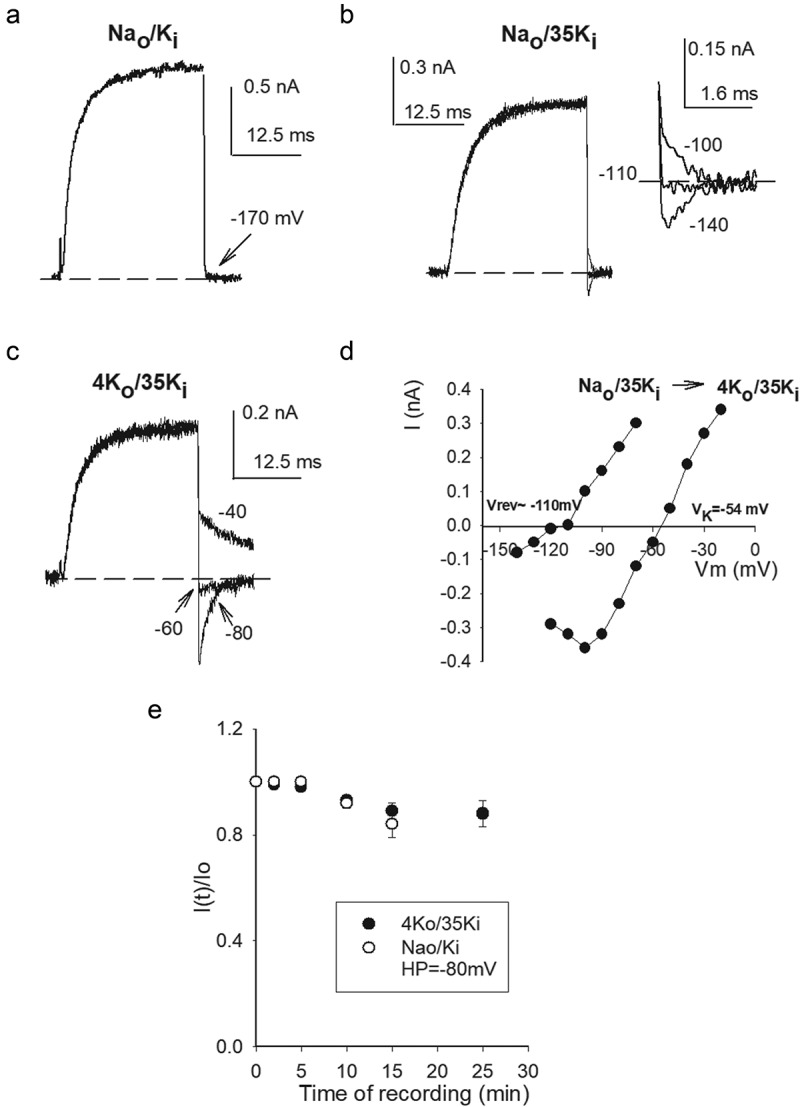
Selectivity and stability in hypokalemic conditions. (a) I_K_ recorded in standard Na_o_/K_i_ solutions. Current was evoked by a 0 mV/30 ms pulse applied from −80 mV; repolarization potential was −170 mV. There is no inward current at −170 mV (b) Superposed currents recorded in Na_o_/35K_i_ solutions in a different cell. Currents were evoked as in A, at pulse end V_m_ was stepped to the potentials indicated in the inset, which shows tail currents in an expanded scale, HP was −120 mV. Note the small but conspicuous inward Na^+^-carried current. (c) Currents recorded in 4K_o_/35K_i_ solutions in the same cell as in B. Tail currents V_m_ are indicated. Notice the shift in current reversal potential as compared to that in B. HP was −120 mV. (d) Instantaneous I–V relationship of the experiments in B &C (see Text). (e) Conductance stability in 4K_o_/35K_i_ solutions. Note that 4 mM K_o_ affords G_K_ stability. Stability in 4K_o_/35K_i_ solutions turned out to be similar to that in standard Na_o_/K_i_ solutions, HP was −80 mV in both cases

In addition to its destabilizing effect on G_K_, we observed that intracellular hypokalemic conditions also drop the pore selectivity, allowing Na^+^ permeation at negative voltages. This is illustrated by comparing currents evoked by a 0 mV/30 ms pulse in cells bathed in a common Na_o_ solution, and internal solutions containing either physiological [K^+^] (Na_o_/K_i_) (Figure 5(a)), or 35 mM [K_i_] (Na_o_/35K_i_, Nernst K^+^ equilibrium potential Vk = −54 mV) (Figures 5(b,c)). In the case of standard Na_o_/K_i_ solutions, at pulse end V_m_ was stepped to −170 mV to provide a strong driving force for, a possible, Na influx through the channels. Note that, as expected for a typical K^+^ channel, there is not any inward, Na^+^-carried current, as indicated by the arrow.In contrast, recordings carried out in Na_o_/35K_i_ solutions (Figure 5(b)), show a conspicuous inward Na^+^ current at −140 mV (easily seen in the Figure inset which shows tail currents in an expanded scale). Note that at −110 mV current is quite small, indicating that this voltage is near the thermodynamic equilibrium potential of the permeant K^+^ and Na^+^ ions. Accordingly, at −100 mV tail current is outward, and carried only by K^+^ ions (see later). It is important to mention that in order to inhibit the ion conductance drop HP was −120 mV. Thereafter, we seek to determine whether physiological K_o_ could restore channels selectivity and/or stability. Thus, once the currents in (Figure 5(b)) were recorded the cell was superfused with 4 mM K_o_ solution. The traces in (Figure 5(c)) (4K_o_/35K_i_) illustrate that 4 mM [K_o_^+^] restores the K^+^ channels characteristic property of excluding Na^+^ permeation, as the current now changes direction at the expected Nernst K^+^ potential (V_K_ = −54 mV). The above is best seen in (Figure 5(d)) which shows the instantaneous I–V relationship of the traces in B&C. Note that: V_rev_(Na_o_/35K_i_)≈ −110 mV, where V_rev_ is the thermodynamic reversal potential (<V_rev_≥ −115 ± 9 mV, n = 5) (see Figure legend, and below); and that on the other hand, V_rev_(4K_o_/35K_i_) = −55.3 mV (<Vrev≥ −55.1 ± 1.5 mV, n = 4). This shows that 4 mM [Ko] halts the anomalous Na^+^ conductance.

**Figure 6.**
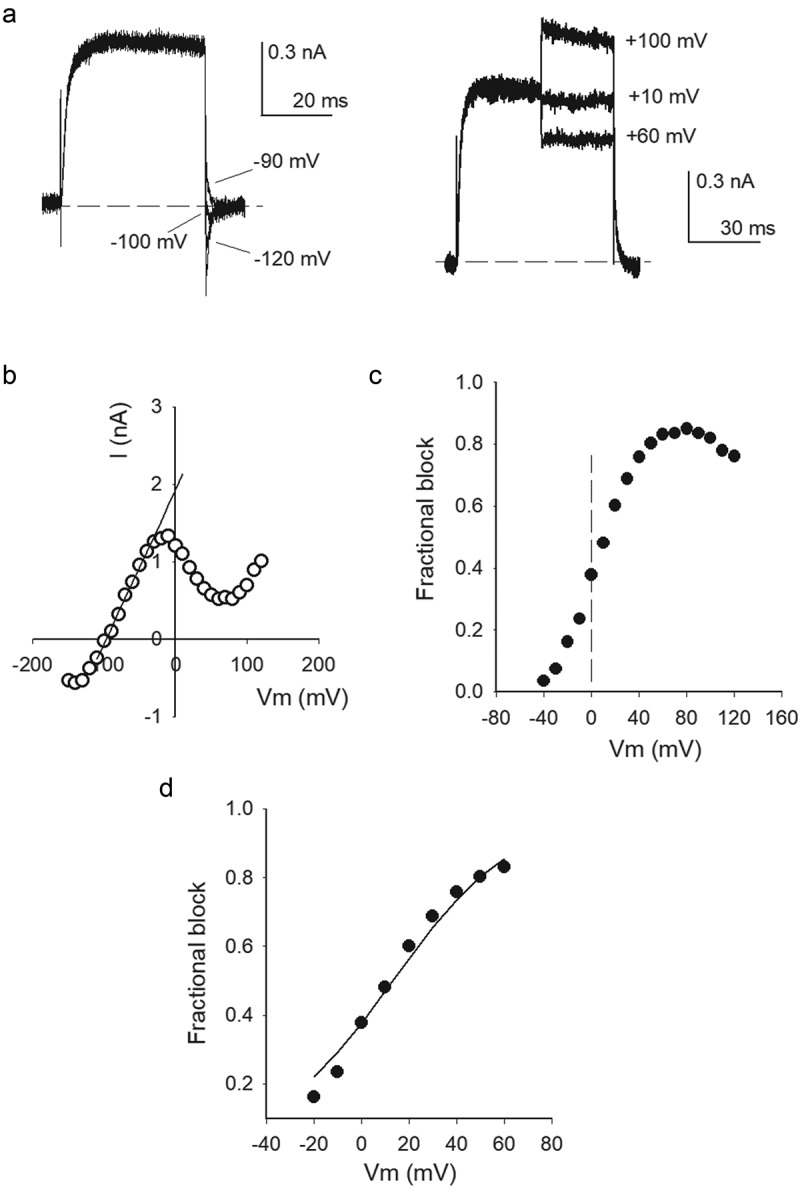
Na^+^ permeation and block in low K^+^ conditions. (a) Left panel: Superposed currents (Na_o_/20K_i_ solutions) evoked by 0 mV/30 ms pulses, at pulse end V_m_ was repolarized to the indicated potentials. Note the inward Na^+^ current. Right panel: currents recorded in the same cell in A illustrating internal Na^+^ block, and the voltage-driven unblock of the pore (see Tex). (b) Instantaneous I–V relationship of the traces in A. (c) Fractional Na_i_ block vs. V_m_. Fractional block was assessed as: f_b_ = 1-(I_o_/I_e_), were I_e_ is the expected current in the absence of Na_i_ block, and I_o_ is the actual, observed, current (see Text). I_e_ was evaluated from the least-squares line (solid straight line through the points in B) that fits the observed current (I_o_) between −80 and −40 mV, where I_o_ varies linearly with V_m_ (r = 0.999): I_e_ = 1.98+(0.019)V_m_. The vertical dashed line helps to visually separate fb at negative vs. positive Vm. (d) Fractional Na^+^ block restricted to the voltage range where f_b_ does not decrease with Vm, data from C. The line is the fit of the points with a Woodhull equation: K_d_(0)*exp[zd(F/RT)*V_m_], with z = +1, d = 0.98, K_d_(0) = 170 mM

**Figure 7.**
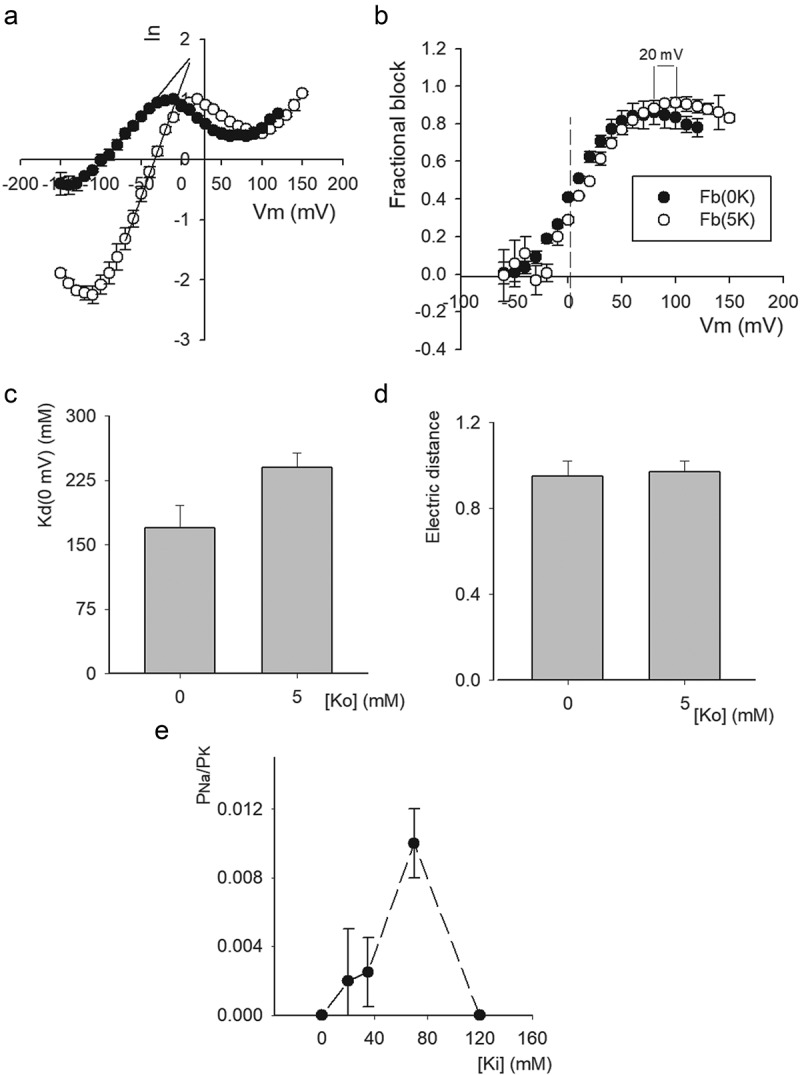
Na^+^ permeation & block with physiological [Ko^+^]. (a) Average instantaneous I–V relationships recorded either in Na_o_/20K_i_ or 5K_o_/20K_i_ conditions obtained from experiments as in Figure 6A. To allow comparison, currents were normalized to its local maximal value (see Text). (b) Fractional Na_i_ block vs. V_m_. Na_i_^+^ block was assessed with the least-squares lines that fit the linear regions of the points at negative Vm, as in Figure 6, namely: 0Ko(−90 to −50 mV): Ie = 1.52 + 0.016*Vm, r = 0.998; 5Ko(−70 to −30): Ie = 1.24 + 0.037*Vm, r = 0.999. The vertical dashed line serves to visually separate negative from positive voltages. (c) Comparison of 0 voltage affinity, Kd(0 mV), in 0 vs 5 Ko conditions; affinities are statistically different (*P* = 0.0634). (d) Comparison of block electrical distance, d, in 0 vs. 5Ko conditions; values are not statistically different (*P* = 0.911). The parameters Kd(0 mV) & d were obtained by fitting the corresponding fractional block vs V_m_ relationships with a Woodhull equation, as in Figure 6. (e) P_Na_/P_K_ vs. [K_i_]. P_Na_/P_K_ was evaluated with the Goldman equation (see Text) applied to the corresponding average current reversal-potentials, from experiments as in Figure 5–7. Points are mean±sem of at least 4 experiments

Although the study of this phenomena falls outside the scope of the present work, notice also that the I–V points with 4 Ko show a conspicuous negative slope at Vm< −100 mV, this slope is caused by external Ca^2+^ block of the pore, its dependence on the ion conditions has already been noticed [6,25] (see also Figures 6 &7).Finally, (Figure 5(e)) shows that, besides halting Na^+^ permeation, 4 mM [K_o_] keeps G_K_ stable. It is interesting to also note that, coincidentally, G_K_ stability in 4K_o_/35K_i_ solutions turned out to be similar to that observed with physiological K_i_ (Nao/Ki), both measured at the HP of −80 mV.

The above observations show that under hypokalemic (non-zero K^+^) conditions G_K_ is destabilized, and Na^+^ permeates through Shab channels at negative voltages. Additionally, notice that the observations collaterally show that a hyperpolarized HP affords G_K_ stability (see also Figure 2(c)) but, as expected, do not restore selectivity. This supports the notion that conductance selectivity and stability are not strictly coupled parameters of K^+^ channels function (see below & Discussion). For completeness, we thereafter assessed Na^+^ interaction with the channels at depolarized voltages, with hypokalemic solutions approaching the 0 K^+^ limit.

### Na^+^ block under near 0 K^+^ conditions ([Na^+^]≫[K^+^] solutions)

It is known that internal Na^+^ blocks K^+^ channels in a voltage dependent manner; under physiological conditions block develops at positive membrane potentials, while still higher voltages relieve Na^+^ block [e.g., 4, 26–30].

Figure 6(a) illustrates the measurement of Na^+^ permeation (negative Vm, left panel), and Na^+^ block followed by pore unblock (depolarized Vm, right panel), carried out in the same cell, with solutions near the 0 K^+^ limit (Na_o_/20K_i_, the lowest [K_i_] which allowed us to find cells with measurable I_K_, with reasonable frequency). The currents in (Figure 6(a)) were elicited by a 0 mV pulse (Figure legend), and thereafter V_m_ was stepped to the indicated potentials; HP was −120 mV to stabilize the ion conductance. The traces in the left show a conspicuous I_Na_ upon membrane repolarization to −120 mV. On the other hand, the traces in the right show that, due to internal Na^+^ block, current at +60 mV is anomalously smaller than that at +10 mV, but that at +100 mV current has an amplitude that is expectedly bigger than that at +60 mV, illustrating the well-known voltage-promoted unblock of the pore [26,27].The above is best seen in the instantaneous I–V relationship of the traces in A (Figure 6(b)), notice that: (a) V_rev_≈ −100 mV. In particular, also notice that both the ionic solutions employed and the I–V plot demonstrate that, for V_m_< V_rev_ inward current is carried only by Na^+^, and that for V_m_ positive to but near V_rev_, outward current is carried only by K^+^ ions, which validates the use of the Goldman bi-ionic equation to asses permeability ratios (see later) (the hint of a negative slope at the most negative Vm is due to Ca_o_^++^ block) (b) Na^+^ block at depolarized Vm becomes apparent at voltages as low as ≈-20 mV, hence from ~-10 to +70 mV the I–V slope is negative, due to the voltage dependence of Na^+^ blockage; (c) at V_m_>+80 mV the I–V slope is again positive, due to the voltage-promoted unblock of the pore [26,27].

The voltage dependence of Na^+^ block of the traces in A is best seen in the fractional Na^+^ block (fb) vs. V_m_ graph (Figure 6(c)). fb was assessed as: fb = 1-(I_o_/I_e_), where I_o_ is the observed current, and I_e_ is the expected current, that would be obtained in the absence of Na^+^ block. I_e_ was evaluated from the least-squares straight-line (solid line in B) that fits the points between −80 and −40 mV, where current varies linearly with V_m_ (see Figure legend), as reported [4]. Notice that: (a) Na^+^ block becomes apparent from negative Vm (e.g., f_b_≈ 20%, at −20 mV). The dashed vertical line serves to point out this feature; (b) Na^+^ block is relieved, fb decreases, at Vm ≥ V_min_≈ +80 mV, where V_min_ stands for the minimum voltage needed to unblock the pore.

Figure 6(d) shows the fb vs. V_m_ relationship of the traces in A, restricted to the voltage range where fb slope is not negative. The line is the least-squares fit of the points with a Woodhull equation (see Figure Legend) with zero-voltage block affinity K_d_(0 mV) = 170 mM, and electrical distance d = 0.95. The latter value suggests that Na^+^ blocks the pore by binding within the selectivity filter itself, as supported by the observations in Figure 5 (see also Figure 7 & Discussion).

The above observations suggest that, in the limit condition of 0 K^+^, internal Na^+^ could possibly interact with those channels that manage to randomly open at the resting potential (see Discussion).

Finally, we tested the effect of physiological [Ko^+^] on Na^+^ block and permeation. Figure 7(a) compares average II–Vs obtained in either Na_o_/20K_i_ (as in Figure 6(b)) or 5K_o_/20K_i_ conditions in 4 different cells. In order to compare both average curves, I–Vs were normalized to its mathematical maximal amplitude (i.e., where the slope of the points is zero). The figure also shows the straight lines Ie, as in Figure 6 (Figure legend). Note that: (1) V_rev_ with 5 Ko is shifted to the expected K^+^ Nernst potential (V_K_ = −35 mV), a shift of ~65 mV compared to V_rev_ with 0 K_o_; and that (2) with both external solutions (0 & 5 Ko) Na^+^ block becomes apparent from negative voltages. Na^+^ blockage in 0 vs 5 mM [K_o_] are best compared in (Figure 7(b)). Block was assessed from the distance of the points to the least squares lines Ie in A, that indicate the expected non-blocked current (see Figure legend). Note that adding 5 K_o_ increases V_min_ in ~20 mV. The latter is in contrast to the 65 mV shift of V_rev_. This shows that V_min_ is a parameter comparatively poorly sensitive to [K^+^] across the membrane. As pointed out by Reuter & Stevens (1980) [30], V_min_ is related to the energy barrier, B_Na_, that has to be overcome for Na^+^ to unblock the pore toward the external solution. B_Na_ can be calculated with the equation [30]: B_Na_ = F*V_min_, where F is the Faraday constant. According to this relationship, adding 5 mM K_o_^+^ increases the energy needed for internal Na^+^ to unblock the pore, in an amount equal to ΔB_Na_ = 1.9 KJ/mol. This extra energy likely arises from the presence of a K^+^ ion dwelling above the Na^+^ blocking site in the pore, with both ions probably located within the selectivity filter.Figure 7(c–d) compare the Na^+^-blockage parameters K_d_(0 mV) and electrical distance d, with either 0 or 5 Ko, respectively, obtained by fitting the data with a corresponding Woodhull equation, as in (Figure 6(d)). Note that, adding 5 mM [K_o_], lefts the electrical distance d basically unchanged (*P* = 0.911), suggesting that, even with 5 mM [Ko^+^] Na^+^ is able to bind within the selectivity filter. On the other hand, see that Na^+^ affinity at zero voltage, K_d_(0 mV), was increased 1.4 times, yielding a ΔΔG = 2.8 J/mol. A change in energy much smaller than that required to unblock the pore ΔB_Na_ = 1.9 KJ/mol.

Finally, (Figure 7(e)) presents the relative Na^+^ permeability (P_Na_/P_K_) of Shab channels bathed in Na_o_ solution, as a function of [K_i_^+^], obtained from experiments as in (Figures 5–7). P_Na_/P_K_ was evaluated by applying the Goldman equation to average thermodynamic current-reversal potentials <V_rev_>, as follows:

P_Na_/P_K_ = [K_i_]/({[Na_o_]*exp(-F*<V_rev_>/RT)}-[Na_i_]); where F, R, & T have their usual meaning.

See that, as internal K^+^ decreases the Na^+^ permeability has a non-monotonic variation, first increasing, but thereafter decreasing as [K_i_] approaches 0, as expected from the conspicuous inward Na^+^ currents under hypokalemic non-0 K^+^ conditions, and from the lack of noticeably Na^+^ currents either with 0 K^+^ across the membrane, or with standard (Na_o_/K_i_) solutions.

## Discussion

Studying Shab channels within the cell membrane environment of cells bathed in 0 K^+^ external solution (Na_o_), we found that as [K_i_^+^] decreases G_K_ drops at −80 mV in a way suggestive of a transition [K_i_], around which the pore either falls relatively fast into an irreversible non-conducting conformation, or remains comparatively longer in a conducting conformation. This observation qualitatively agrees with studies carried out with isolated KcsA channels, where a transition [K^+^] was suggested, around which two structures are formed, either the so called low (non-conducting) or high K^+^ (capable to conduct), crystal structures [12–14]. It is important to note however that in Shab even when G_K_ is comparatively stable with 120 > [K_i_] ≥ 60 mM, the pore structure is nonetheless somehow altered, as evidenced by the anomalous permeation of Na^+^ at negative voltages.

On the other hand, comparison of the effect of non-saturating, nearly mirror, [K^+^] across the membrane on conductance stability, showed that K^+^ effect is asymmetrical, with external K^+^ being significantly more effective than internal K^+^. This asymmetry could simply be a consequence of the externally oriented location of the selectivity filter, and/or of the cytoplasmic location of the activation gate, which sets a barrier to K^+^ flux [9,31]. It could also obey to a differential stabilizing role of the different K^+^-sites of the pore (see below). Whatever the case, the asymmetric effectiveness of K^+^ stands as a physiologically relevant feature of K^+^ channels functioning.Regarding the above, we found that the potency with which external cations prevent Shab G_K_ drop (K^+^≈Rb^+^>Cs^+^>NH_4_^+^≫Na^+^) does not match the K^+^ channels selectivity sequence. We hypothesize that the G_K_ protection-sequence may correspond to the selectivity of the particular K^+^-site(s) where G_K_ is kept stable by externally present suitable ions (see below for TEA). As a reference, it should be mentioned that monovalent ions stabilize the KcsA tetramer with a potency equal to that here reported for Shab [32,33]. Although, on the other hand, in Shaker*B*, it was found that all permeant ions protect G_K_ with comparable effectiveness [2]. The structural basis of this difference is puzzling since both channels share the conserved signature-sequence of K^+^ channels.

In addition to permeant ions, we observed that the, non-permeant, TEA ion protects G_K_ from the external side of the membrane. This result agrees with observations showing that TEA_o_ inhibits G_K_ drop of Shaker in 0 K^+^ [2]. In the case of Shab, but not of Shaker, in order for TEA_o_ to efficiently protect G_K_, it was necessary to add a low (0.5 mM) [K_o_^+^], a K^+^ concentration which by itself does not inhibit G_K_ drop. K^+^ was added to the TEAo solution after considering that mammalian Kv2.1 channels are blocked by external TEA with a Kd of 5 mM [34,35], but cease to be blocked by this cation in the absence of K^+^. The latter occurs because a lysine residue (K326) in the outer vestibule of Kv2.1 pore seems to reorient in a way that fully hinders TEA_o_ binding in the absence of K^+^ [22,34]. Shab also has lysine at the equivalent Kv2.1 position, which along with the small extent of TEA protection of G_K_ observed in the absence of external K^+^, suggests that this residue may undergo a similar reorientation in 0 K^+^ in both channels.

Interestingly, internal TEA also ceases to block Kv2.1 in the absence of K^+^, this shows that the conformational channel triggered from the lack of K^+^ extends from the external vestibule to the central cavity of Kv2.1. Pore block by both internal and external TEA are equally restored by the low micromolar amounts of K^+^ which also block Na^+^ permeation through Kv2.1 [34].

In the case of Shab, and the squid K channel, pore block in Nao/Ki solutions, by either internal quinidine, or TEA, promotes the irreversible collapse of G_K_ [36,37]. This effect arises because cation-blockers (like quinidine and TEA), upon binding within the central cavity, electrostatically repel the stabilizing K^+^ ions dwelling in the selectivity filter, leading to a virtual 0 K^+^ condition where Gk irreversibly collapses.

As mentioned before, besides abolishing TEA block it was also observed a flux of Na^+^ through Kv2.1 in the absence of K^+^ [22,34], however, we are not aware of any studies regarding the K^+^-dependent stability of Kv2.1 channels.

TEA_o_ inhibition of Shab G_K_ drop, along with the significant asymmetry of K^+^ effectiveness to maintain G_K_ stable, suggest that the outermost pore site (s1/s2), and/or a conformational rearrangement of the external pore vestibule, have a relevant role regarding G_K_ stability (see also [38]).

### Na^+^ permeation and blockage with hypokalemic solutions

In addition to destabilize G_K_, hypokalemic (non-zero K^+^) solutions allow the passage of Na^+^ through Shab. Na^+^ permeation in the absence of K^+^ has been observed in other K^+^ channels, as for example Shaker [38–40], Kv2.1 [22], and Kv1.5 [41]. However, this has not been observed in all K^+^ channels, as for example Kv10.1 (unpublished observations). Here we showed that, adding low, physiological [K_o_] restores both selectivity and G_K_ stability.

As [K_i_] decreases the relative P_Na_/P_K_ ratio reaches a peak and thereafter decreases, as [K_i_] approaches 0. This behavior underlines the lack of a conspicuous Na^+^-current in the limit of 0 K^+^. Thus, although K^+^ prevents Na^+^ permeation, likely through a Koshland induce-fit mechanism [22,42], a minimal [K^+^] is nonetheless required to keep a pore conductive-structure that, paradoxically, allows Na^+^ permeation. The non-monotonic P_Na_/P_K_ variation contrasts with the progressive (monotonic, although non-uniform) G_K_ stability decrement as [K_i_^+^] decreases. This difference, along with the differential effect of hyperpolarized V_m_ on G_K_ stability and selectivity, further supports the notion that: pore stability and selectivity, although clearly related, are not strictly coupled parameters (see also [6,8]).

With solutions that approach the 0 K^+^ limit (Na_o_/20K_i_), we observed that Na^+^ block becomes apparent from negative potentials, with a blockage electrical-distance near to one. These observations: (a) reinforce the notion that Na^+^ ions are indeed capable to bind within the selectivity filter itself; (b) suggest that in the limit of 0 K^+^ across the membrane Na^+^ may possibly interact with those channels that manage to randomly open at the resting potential, somehow catalyzing G_K_ collapse. This hypothesis may explain at least part of the stabilizing effect afforded by hyperpolarized potentials, since hyperpolarized Vm values further prevent channel opening, hence they should also decrease Na^+^ block of randomly open channels (for an extensive discussion of the role of V_m_ in G_K_ stability see [7,8,38]).

### Summary of Shab G_K_ stability and selectivity under hypokalemic solutions

**Figure 8.**
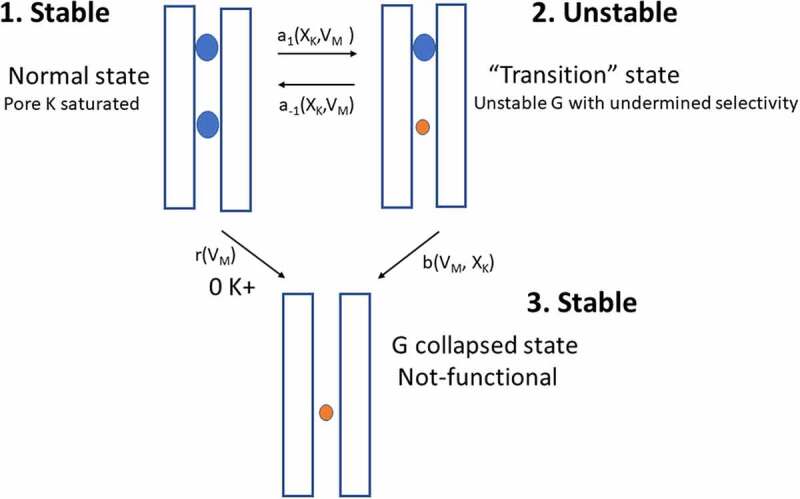
Summary of Shab K^+^ channel behavior as a function of [K^+^] across the membrane. Upper left image (state 1): Under saturating [K^+^] the selectivity filter has 2 K^+^ ions at a time (blue spheres, Doyle et al [9]), Gk is stable & selective, irrespective of Vm. Upper right panel (state 2): Upon K^+^ replacement by equimolar amounts of Na^+^ ions the channel visits a “transition”, meaning unstable, state (1->2, right panel), where Gk falls with a rate that depends on X_K_ & Vm (K^+^ molar fraction) and Vm. In this unstable state the pore conformation allows Na^+^ permeation from the open, non-inactivated, state (a Na^+^ ion is indicated in the pore by the small red sphere), depending on Vm. Negative HP or K^+^ addition restore stability (indicated by the left pointing arrow), although: the already lost G is not recovered (i.e., arrows connecting states 1 & 2 do not indicate a truly reversible transition). Lower panel (state 3): With 0 K^+^ across the membrane (1->3) the channel readily falls into the stable (i.e., irreversible) not conducting state 3; alternatively, under hypokalemic (non-zero K^+^ conditions) the channels sink although more slowly into state 3 (2->3) depending on X_K+_. In both cases (1->3 and 2->3) the rate of Gk collapse depends on Vm. In the nonconducting 0 K^+^ state 3 the pore may possibly contain Na^+^ ion(s), but it is unable to conduct any type of ion

The observations herein reported are summarized in cartoon form in (Figure 8). The upper left panel (labeled as state 1) depicts the stable and K^+^ selective selectivity filter of the pore, as seen in high K^+^ (saturating) conditions ([9]), (a condition seemingly reached in either Nao/Ki, or 30Ko/Nai solutions). There are two K ions at a time in the filter (blue spheres) ([9]).

Upon partly replacing K^+^ with Na^+^, channels transit to an unstable “transition” state (labeled 2), having an undermined G_K_ stability and selectivity, at a rate a1 that depends on both X_K_ and Vm, as indicated. In the unstable state 2 the pore allows Na^+^ permeation at negative Vm, hence a Na^+^ ion is depicted in the selectivity filter as a small red sphere, and G_K_ spontaneously drifts to zero (state 3), depending on Vm and X_K_. The unstable state 2 can be only partly reverted, and the channels regain stability and selectivity, by actively adding K^+^, indicated by a-1. However, the fraction of already lost G_K_ (transition 2 -> 3), cannot be recovered.

The non-conducting and irreversible state 3 can be reached directly, and comparatively faster, from the stable high K^+^ condition (1->3) upon full removal of K^+^, at a rate r that depends on Vm. As a hypothesis we depicted the stable (irreversible) state 3 as containing a Na^+^ ion(s) within the selectivity filter, but without being capable of conducting any type of ions. Finally, it must be commented that the 2TM KcsA tetramer losses its thermal stability in the absence of K^+^ [32,33], this possible final step, that would follow state 3, has not yet been demonstrated in 6TM K^+^ channels.It is important to point out that in sate 2 Shab allows the passage of Na^+^ from the open (non-inactivated) state (as evident in the traces in Figures 5–6). This differs from Shaker channels which allow the transient passage of Na^+^ from the C-type inactivated state, before collapsing (see Figure 1 of Hoshi & Armstrong, 2013 [38], and references therein cited), and in this case in a reversible manner [2]. This difference may somehow be related to the fact that Shab slow inactivation is not a C-type inactivation [43], see below.

### Physiological consequences of the K^+^-dependent stability of K^+^ channels

Although the number of K^+^ channels whose possible K^+^-dependent stability has been study is still small, conditions in which G_K_ loss by hypokalemia may be related to a pathological condition had already been reported, for example:As mentioned before, intracellular channel blockers of clinical use, like the antiarrhythmic quinidine may cause the G_K_ collapse of delayed rectifier K^+^ channels, like Shab or the squid channel, under, extracellular, hypokalemic conditions [36,37]. Within the organism the latter condition can be elicited by a number of factors, including vomiting [44]. The drop of G_K_ being a risky, potential side effect, of intracellularly-acting cationic drugs [36,37]. Regarding heart physiology, it has been reported that with a low serum [K^+^] the HERG K channel, a key participant of the repolarization phase of the cardiac action potential, and hence of cardiac rhythm, cease to conduct K^+^ by falling in a reversible non-conducting state, a condition that can lead to sudden cardiac death [45,46]. Thereafter, non-conducting HERG channels are removed from the cardiac muscle membrane, so when the normal serum [Ko^+^] is reestablished by hemostatic mechanisms, cardiac muscles have a transiently lower HERG channels density, with a risk of developing arrythmias [45,46]. HERG channels fall into this non-conducting state in a gating-independent fashion, in a manner seemingly similar to that of Kv1.4 channels [45–47]. These examples show that more studies are still needed to assess the possible K^+^-dependent stability, and selectivity, of K^+^ channels subtypes. Finally, for the sake of completeness, it should be mentioned that increases in extracellular K^+^, like those that transiently occur in the confined extracellular medium of neurons upon firing action potentials, produce the well-known acceleration of recovery from ball-and-chain inactivation, and slow down the entry into C-type inactivation ([38] and references therein cited). Shab presents a non-C type slow inactivation, increases of external K^+^ accelerate the entry [43] and slow down the recovery from slow inactivation [48]. These examples show that increases in external [K^+^] dynamically change the amount of K^+^ channels available to repolarize the cells.
